# Report on the 1st scientific meeting of the "Verein zur Förderung des Wissenschaftlichen Nachwuchses in der Neurologie" (NEUROWIND e.V.) held in Mittenwalde/Motzen, Germany, Oct. 30th - Nov. 1st, 2009

**DOI:** 10.1186/2040-7378-2-7

**Published:** 2010-03-31

**Authors:** Tim Magnus, Ralf Linker, Sven G Meuth, Christoph Kleinschnitz, Thomas Korn

**Affiliations:** 1 Department of Neurology, University Clinic Hamburg-Eppendorf, Martinistr. 52, D-20246 Hamburg, Germany; 2 Department of Neurology, St. Josef-Hospital, Ruhr-University, Universitätsstraße 150, D-44801 Bochum, Germany; 3 Department of Neurology, University of Würzburg, Josef-Schneider-Str. 11, D-97080 Würzburg, Germany; 4 Department of Neurology, Technical University of Munich, Klinikum rechts der Isar, Ismaninger Str. 22, D-81675 Munich, Germany

## Abstract

Report on the 1st scientific meeting of the "Verein zur Forderung des Wissenschaftlichen Nachwuchses in der Neurologie" (NEUROWIND e.V.) held in Mittenwalde/Motzen, Germany, Oct. 30th - Nov. 1st, 2009

A scientific meeting report

## Introduction

It is with great pleasure and enthusiasm that we introduce the new non-profit "Association for Supporting Young Scientists in the Field of Neurology in Germany" ("Verein zur Förderung des Wissenschaftlichen Nachwuchses in der Neurologie", NEUROWIND e.V.) http://www.neurowind.de. As its name suggests, the association is intended to promote the work of young neurologists and neuroscientists in German-speaking European countries. Founded by the neurologists Ralf Linker, Bochum, Thomas Korn, Munich, Tim Magnus, Hamburg, Sven G. Meuth, and Christoph Kleinschnitz, Würzburg, Germany, NEUROWIND e. V. aims to provide an interdisciplinary and interactive platform for young researchers in order to gather and disseminate new knowledge in the fields of clinical and basic neurosciences. NEUROWIND e. V. focuses on three main topics: [1] cerebrovascular diseases, [2] neuroinflammation, and [3] neurodegeneration.

Tremendous progress has been made in recent years in understanding the pathophysiology of individual disease conditions such as multiple sclerosis (MS), stroke, or Alzheimer's disease (AD). In spite of this success in unravelling disease mechanisms, the translation of novel experimental therapies into effective treatment for patients has so far been unsatisfying for a number of reasons, with one central problem certainly emanating from insufficient stringency of the translational process from bench to bedside and vice versa. One clear aim of NEUROWIND e. V. is therefore to bring together basic pathological and therapeutic concepts from different neurological disease models which at first glance might appear largely unrelated. However, numerous studies meanwhile have taught us that pathophysiological pathways and effector mechanisms appear to be common to a variety of different diseases. Good examples are the only recently recognized role of inflammation in stroke, M. Parkinson and AD or degenerative processes during the course of MS. Our ultimate goal is to overcome out-dated model barriers and raise the efficacy and quality of translational neurological research by fostering exchange of information and providing an interactive platform that favors fruitful discussions necessary to identify and solve distinct problems.

To reach these ambitious goals, the first scientific meeting of NEUROWIND e.V. was recently held from Oct. 30'th - Nov. 1'st, 2009 in Mittenwalde/Motzen, Germany. Approximately 60 participants mainly on the level of doctoral students and young postdocs joined the meeting and presented their scientific work in the beautiful and stimulating environment of the Prussian scenery. A brief summary of the most interesting findings from the five sessions is given below and the full program as well as further information is available at http://www.neurowind.de.

## Summary of the scientific contributions to the NEUROWIND meeting 2009

### Contributions in the fields of neuroimmunology and neurodegeneration

Experimental autoimmune encephalomyelitis (EAE) is widely used to investigate the biology of autoreactive T cells in vivo. While EAE is considered an animal model to simulate inflammatory aspects of MS, similar aspects can be studied in the peripheral nervous system in the model of experimental autoimmune neuritis (EAN; work on new therapeutics in EAN presented by Gerd Meyer zu Hörste from the group of Bernd Kieseier, Dept. of Neurology, University of Düsseldorf). A series of myelin antigens have been identified as potential targets of autoreactive T cells and the most frequently used epitope to induce EAE in C57Bl/6 mice is the myelin oligodendrocyte glycoprotein (MOG) peptide 35-55. T cell receptor (TCR) transgenic mice with specificity for MOG35-55 have been generated and are used as a spontaneous model of MS since up to 10 percent of MOG35-55 TCR transgenic mice develop EAE without active immunization. As has recently been found by the group of Hartmut Wekerle and Florian Kurschus (Max Planck Institute for Neurobiology, Munich and Institute of Molecular Medicine, University Medical Center of the Johannes Gutenberg-University Mainz), MOG35-55 specific T cells from TCR transgenic mice also recognize another autoantigen, i. e. neurofilament-M (NF-M) peptide 18-30 [1]. Although NF-M and MOG belong to completely distinct protein families, the NF-M epitope shares essential TCR-contacting residues with MOG35-55 that allow for it to be recognized by MOG35-55 specific T cells when bound to the C57Bl/6 MHC class II complex (I-Ab). From these studies arises the concept of 'cumulative' autoimmunity with a degenerate TCR receptor recognizing several autoantigens that may not be related, thus surpassing the threshold to clinically manifest organ specific autoimmune inflammation. In a variety of EAE models using knockout animals, factors that enhance the pathogenicity of autoreactive T cells were characterized.

In the past, IFN-γ production by autoreactive T cells has been regarded as pathogenic hallmark of autoantigen specific T cells, which prompted the idea that organ specific autoimmunity might be a "Th1 disease" [2]. More recently, another phenotype of CD4+ T helper cells, so-called Th17 cells have been implicated in the development of EAE and other autoimmune disorders [3]. However, the plasticity of Th17 cells, which have been named after their signature cytokine IL-17, appears to be greater than that of Th1 cells. Whereas the combination of TGF-β and IL-6 in mouse (and TGF-β plus IL-21/or IL-1 in man) are the differentiation factors for Th17 cells [4-7], IL-23 has a major role in the stabilization of IL-17 production by Th17 cells and thus in stabilization of the functional phenotype of these cells. Very recent work performed in the group of Thomas Korn (presented by Malte Christian Claussen, Dept. of Neurology, Technical University of Munich) points to functions of IL-23 that are extrinsic to classical αβ T cells since non-classical T cells and perhaps even non-T cells express the IL-23R and respond to IL-23 by production of cytokines. Usage of IL-23R reporter mice will provide us with important information on the role of IL-23 activated non-classical T cells in tissue inflammation and autoimmunity. The population dynamics and migration events of both T cells and non-T cells into the CNS during EAE are still under intense investigation, and meticulous flow cytometric analysis has been applied by Karin Steinbach from the groups of Manuel Friese and Eva Tolosa (Center for Molecular Neurobiology, Hamburg) to reveal the temporal pattern of immune cell infiltration into the CNS during EAE. At the onset of disease, Th1 cells and Th17 cells accumulate in the CNS almost simultaneously. However, IL-17 production by T cells in the CNS appears to be sustained in actively induced MOG35-55 EAE. As far as the T cell/target interaction in the CNS is concerned, still little is known about the relative impact of Th1 vs Th17 cells. Indeed, the exact pathogenic role of Th17 cells in vivo is not yet understood. Besides secreting cytokines and chemokines to attract other immune cells like neutrophils, Th17 cells may also directly interact with target structures in the CNS like naked axons that have been stripped off their myelin sheaths. Here, exciting in vivo imaging studies using the two photon technique [8] have been presented by Volker Siffrin from the group of Frauke Zipp (Dept. of Neurology, University Clinic of Mainz). These investigations revealed that Th17 cells indeed were able to induce axonal damage in an MHC class II independent manner. Although the mechanism of lesion generation remains to be determined, perforin degranulation may be involved in inducing axonal damage.

While the role of CD4+ T cells for the induction of immunopathology is a proven fact in EAE, it has been difficult to assess the mechanism of how CD8+ cytotoxic T cells contribute to lesion formation in this model. Interestingly, CD8+ T cells are present in human MS lesions and have been shown to be clonally expanded suggesting a pathogenic role of these cells [9]. However, only recently new tools have become available to study the role of CD8+ T cells in an experimental setting [10]. Furthermore, double transgenic mice have been generated that express OVA as a neo-autoantigen in a cell type specific manner in oligodendrocytes under the MBP promoter and at the same time bear a transgenic TCR for a specific OVA-peptide on their CD8+ T cells (OT-I) [11]. In a highly interesting study by Kerstin Göbel and Nico Melzer from the group of Heinz Wiendl (Dept. of Neurology, University Clinic of Würzburg), brain slices from MBP-OVA transgenic mice have been used to investigate the interaction of OT-I cells with oligodendrocytes presenting OVA-peptide [12]. While OT-I cells induced apoptosis in oligodendrocytes via direct attack, neighboring neurons were also affected, revealing the possibility of perforin-driven collateral damage in neurons. Besides immunohistochemical and optical methods to characterize lesion formation on a molecular level in vivo, electrophysiological methods like patch clamp approaches are also being used to further characterize the impact of cytotoxic T lymphocytes (CTLs) on neurons. In vitro, it is possible to measure the break-down of the neuronal membrane potential when these neurons, that have been induced to express MHC class I and have been loaded with OVA peptide, are attacked by OVA-peptide specific CD8+ OT-I T cells. Again it appears that this process of CTL-dependent damage to neurons is perforin mediated since perforin deficient OT-I cells fail to short-circuit axons. In order to approach questions of CTL/target interaction on the molecular level in vivo, viral models of neuroinflammation are helpful tools. Recently, a model of LCMV infection is being explored in which the infecting agent (an RNA virus) can be manipulated by reverse genetics and specific T cell responses against infected neurons can be monitored [13,14].

In this model which is being established by Mario Kreutzfeldt from the group of Doron Merkler at the Dept. of Neuropathology, University of Göttingen, the avidity of the CTL/peptide/MHC class I interaction can be modified and differential immunopathological responses can be studied in vivo. Lesion generation in neuroinflammation is not only dependent on cytotoxic CD8+ T cells, but monocytes and macrophages are major players in inducing damage to myelin and neurons. While T cell-derived IFN-γ is considered the canonical molecule to activate macrophages, a series of other stimuli can trigger effector functions in these cells. Interestingly, adhesion of monocytes to immobilized platelets results in massive TNF production by monocytes. This phenomenon is now being characterized by Harald Langer in the group of Triantafyllos Chavakis (NIH, Bethesda, USA). The molecular basis for the interaction of platelets and monocytes appears to be a ligand/receptor interaction between GPIb on platelets and Mac-1 (CD11b/CD18) on monocytes. Blockade of this interaction results in diminished secretion of TNF by macrophages, and GPIb deficient mice develop attenuated EAE suggesting that platelet-mediated activation of macrophages might be an important effector mechanism in this disease. Besides immune cell/neuron interactions, several further pathways may play an important role in inflammation-mediated neurodegeneration. Among others, neurotrophic factors and ion channels have recently been in the focus of interest. Neurotrophic factors comprise neutrophins and neurotrophic cytokines which are mainly produced in the nervous system, but also by immune cells. As a prototype mediator, the role of brain derived neurotrophic factor (BDNF) and its receptors trkB and p75NTR have been characterized in MS lesions and more recently also in EAE models [15-17]. Using an experimental approach with bone marrow chimera, Tobias Dallenga from the groups of Stefan Nessler and Christine Stadelmann (Dept. of Neuropathology, University of Göttingen) has studied p75NTR-mediated signaling in immune cells and non-immune cells during EAE. These data suggest an important role of immune cell-derived BDNF and p75NTR-mediated signaling pathways for axon protection. In autoimmune inflammation, Nav and Kv channels as well as acid sensing ion channels were all shown to play a role for axon or glial cell function but in part also for regulation of the immune cell response [18,19]. More recently, a new family of genes encoding two-pore domain potassium channels that generate "leak" potassium currents were characterized. The TASK subfamily of channels, notably TASK-1 (KCNK_3_) and TASK-3 (KCNK_9_), have now been shown to modulate inflammation and neurodegeneration in EAE and probably also ischemic stroke, thus identifying new potential molecular targets for the therapy of inflammatory and degenerative CNS disorders. These data were presented by Petra Ehling from the group of Thomas Budde, Dept. of Physiology, University of Münster.

Investigation of immune cell/target cell interactions is certainly a major domain of animal studies. Yet, studies with human immune cells are required to test the relevance of hypotheses that have been raised in animal models. We are now witnessing the first reports on Th17 cells in human MS on a larger scale. Notably, the frequency of Th17 cell clones but not Th1 clones in the peripheral blood and the CSF appears to be correlated with disease activity in relapsing remitting MS [20,21]. In ex vivo analyses performed by Verena Brucklacher-Waldert from the group of Eva Tolosa (Center for Molecular Neurobiology, Hamburg), Th17 cells had a strongly activated phenotype and were relatively resistant to regulatory T cell (Treg)-mediated suppression in comparison with Th1 cells. In contrast to EAE which is clearly dependent on CD4+ T helper cells, human MS is more complex and a plethora of other immune cells are directly or indirectly involved in the generation of MS lesions. NK cells have been of particular interest in this regard since their modulation appears to be the basis of the efficacy of a monoclonal antibody to the IL-2R (daclizumab) that has recently been tested in clinical trials [22]. Brady Messmer from Jan Luenemann's group at the Institute for Experimental Immunology, University of Zuerich, Switzerland studied the role of NK cells in MS patients in more detail. NK have been identified in MS lesions. CD56^dim ^NK cells are equipped with the molecular machinery to kill their target cells and are more prominent in PBMCs as compared with lymph nodes. In contrast, CD56^bright ^NK cells produce cytokines like IFN-γ, TNF, and GM-CSF and are more abundant in lymph nodes than in PBMCs [23]. CD56^bright ^NK cells are divided into CD16^- ^and CD16^+ ^populations. In order to evaluate the role of NK cells in the pathogenesis of MS in more detail, NK cells of MS patients were studied and compared with those of healthy control subjects. In MS patients, CD56^bright^CD16^- ^NK cells show impaired expansion and produce less IFN-γ in response to IL-12 whereas the lytic function of NK cells (CD56^dim^) appears to be unchanged. These data suggest that subsets of NK cells in MS patients display a functionally different phenotype and may be implicated in the disease process.

Further lessons on the relation between immune cells and CNS tissue can be learned from degenerative diseases and their respective animal models. Here, studies in mouse models of Alzheimer's disease (AD) as well as Huntington's disease (HD) recently gained much interest. Initially, AD has been characterized by formation of amyloid plaques, neurofibrillary tangles and subsequent neurodegeneration. More recently, the role of immune cells, most notably microglia, in this process has been characterized. While the exact sequence of events eventually leading to neuronal death still remain to be determined, several mediators involved in regulation of microglia have been identified. Here, especially chemokines and their receptors were found to play an important role, e.g. for cell migration (work presented by Marius Krauthausen from the group of Markus Müller and Michael Heneka, Dept. of Neurology, University of Bonn). The modulation of such factors may critically regulate microglial function and finally also influence the process of neurodegeneration. Similar observations were reported in models of HD which are characterized by formation of intraneuronal huntingtin aggregates and neuronal dysfunction. Here, innovative neurobiological treatment approaches such as anti-sense technologies or stem-cell based repair strategies can reduce huntingtin aggregate load and improve functional deficits in mouse models of HD as presented by Christian Saß from the Dept. of Neurology, University Clinic of Aachen. In addition, more established treatment strategies such as therapy with immunomodulators appear to be effective as well (Christiane Reick, Dept. of Neurology, St. Josef-Hospital, Ruhr-University Bochum). These data point to a role of the immune system in HD, but also to a putative neuroprotective function of these drugs, thus opening up an exciting new avenue of translational research linking the fields of neuroimmunology and neurobiology.

### Contributions on stroke and vascular pathology

Ischemic stroke is a devastating disease that represents the second leading cause of death worldwide. Each year, 575.000 people in Europe fall victim to ischemic stroke, which is estimated to cost 71.8 billion Euros [European Stroke Initiative]. It is estimated that the lifetime risk for stroke is between 8% and 10%. Early restoration of blood flow remains the treatment of choice for limiting brain injury following stroke. While reperfusion of the ischemic brain is desirable in principle, it may also foster tissue damage under certain conditions. Reperfusion appears to augment the inflammatory response and causes additional injury to adjacent brain tissue. Hence, a rapidly evolving area of stroke research involves defining the molecular and cellular basis for this secondary tissue injury and inflammation associated with transient cerebral ischemia. For this research, primarily the middle cerebral artery occlusion reperfusion model in mice is used.

The inflammatory response seen in this model is initiated by an accumulation of microglia and the secretion of pro-inflammatory cytokines such as IL-1β, IL-6 or MCP-1 (researched by Mathias Gelderblom from the groupd of Tim Magnus, Dept. of Neurology, University Clinic Hamburg-Eppendorf, Hamburg). On the cellular level, infiltration of the ischemic hemisphere by macrophages, lymphocytes, and dendritic cells (DCs) within the first day precedes neutrophilic influx. Up-regulation of MHC-II and the co-stimulatory molecule CD80 demonstrate activation of these cells arguing for a pro-inflammatory environment. However, also regulatory immune cells (NKT cells, CD4-/CD8-T lymphocytes, Foxp3+ T cells) accumulate in the ischemic brain [24].

The functional relevance of inflammatory cells can be proven in knockout animals such as Rag deficient mice that lack B and T cells and are largely protected from inflammatory damage secondary to ischemic stroke [25]. Conversely, depletion of regulatory T cells (Treg) induces a significant increase in infarct size pointing to a relevant part of these cells in regulating post-stroke inflammation [26]. Furthermore, inhibition of cell migration into the lesioned brain might become an interesting approach to modulate stroke-induced immune pathways. Arthur Liesz from the group of Roland Veltkamp, Dept. of Neurology, University of Heidelberg, showed that blockage of immune cell entry results in smaller infarcts and an improved neurological outcome. This blockage was achieved by an antibody preventing the binding of α 4 integrins, a common therapeutic approach also in MS. So far, it remains unclear which cell type (if any) is the key player in ischemic stroke. However, it seems likely that T cells and their subtypes play an important role while the function of neutrophils, which also express α 4 integrins, is not yet clear.

In contrast to the local pro-inflammatory response within the CNS, changes in the systemic immune compartment indicate a more general stroke-associated immune suppression. The latter can, as Odilio Engel from Andreas Meisel's group at the Dept. of Experimental Neurology, Charité Universitätsmedizin Berlin, points out, be observed in patients as well as in the animal model, where an increased bacterial load is found in the lungs of stroked rodents. The immunosuppressive effect may be elicited by an increase in vagal activation and subsequent secretion of acetylcholine in lymph nodes and spleen.

Another facet in stroke research is related to the occurrence of oxidative stress. Cells and especially neurons have to deal within minutes with reactive oxygen and nitrogen species (ROS/RNS). One attractive candidate source for oxidative stress in acute ischemic stroke are NADPH oxidases, the only known enzyme family that has ROS as their sole enzymatic product. These are the molecules of a specific research interest for Tobias Schwarz form Christoph Kleinschnitz's group at the Dept. of Neurology, University Clinic of Würzburg. In rodents 4 NOX genes exist, and in the rodent brain NOX are mainly expressed in neurons and the vasculature with NOX4 being the most abundant isoform. In an interesting study using NOX1, NOX2 and NOX4 deficient mice as well as the specific NOX Inhibitor VAS2870, the pathophysiological role of the different NOX isoforms in ischemic stroke has now been assessed in terms of infarct development and blood-brain-barrier damage.

Our current performance in the acute treatment of stroke patients is moderate at best and, therefore, additional efforts to enhance tissue repair are badly needed. As outlined above, inflammatory cascades are active during cerebral ischemia. However, their effects are not necessarily detrimental, as Karen Gertz from the group of Matthias Endres, Dept. of Neurology, Charité Universitätsmedizin Berlin, reminds us. IL-6, for example, helps to increase vascular repair and possibly neogenesis and can improve long term outcome in experimental stroke. Other strategies involve the use of stem cells (presented by Jens Minnerup from the group of Wolf-Rüdiger Schäbitz, Dept. of Neurology, University of Münster). However, it appears that significant tissue repair derived from endogenous stem cells is not realistic in ischemic stroke, at least in the near future. The systemic application has turned out difficult since it is not easy to derive the perfect cell that is undifferentiated enough to integrate and survive but develops into a functional neuron. Although some functional improvement can be seen in stroked rodents after the application of neurospheres depending on their differentiation protocol, the therapeutic effects are still relatively small. Finally, Jan Klohs from Ulrich Dirnagl's group at the Dept. of Experimental Neurology, Charité Universitätsmedizin Berlin, introduced a new imaging technique in rodent stroke models [27]. Near-infrared fluorescence (NIRF) imaging is suitable to visualize distinct molecules involved in the pathophysiology of ischemic stroke in rodents in vivo by utilizing specific NIRF probes, e.g. against matrix metalloproteinases (MMPs). Although its temporal resolution is still relatively low, this non-invasive method could be useful in monitoring treatment responses in individual animals over time.

### Concluding remarks

Lesion formation and repair are always essential events in pathological conditions in the CNS. Although distinct diseases, vascular, inflammatory and neurodegenerative disorders of the CNS may share pathological sequences on the cellular and molecular level. Decades of experience and gathering of knowledge in the formation, trafficking and effector functions of immune cells in neuroinflammatory conditions may help to better understand the involvement of immune cells in vascular diseases and neurodegenerative disorders. Thus, comparing cellular reactions in the peripheral immune compartment and the CNS across different disease models may offer an unconventional but very efficient means to generate new ideas and promote research. We hope that our initiative will help young researches to create new concepts and opportunities to improve the understanding of many neurological diseases and to find new treatment options. We encourage all members of the neurological community to support our idea and provide constructive input for the upcoming meeting in October 2010.

## Competing interests

The authors declare that they have no competing interests.

## Authors' contributions

TM, RL, SGM, CK and TK wrote the paper. All authors read and approved the final manuscript.
